# Differential regulation of meristem size, morphology and organization by the ERECTA, CLAVATA and class III HD-ZIP pathways

**DOI:** 10.1242/dev.129973

**Published:** 2016-05-01

**Authors:** Tali Mandel, Héctor Candela, Udi Landau, Lior Asis, Einat Zelinger, Cristel C. Carles, Leor Eshed Williams

**Affiliations:** 1 The Robert H. Smith Faculty of Agriculture, Food and Environment, The Hebrew University of Jerusalem, POB 12, Rehovot 76100, Israel; 2 Instituto de Bioingeniería, Universidad Miguel Hernández, Campus de Elche, Elche 03202, Spain; 3 Université Grenoble Alpes, Laboratoire de Physiologie Cellulaire et Végétale (LPCV), Grenoble F-38054, France; 4 CNRS, LPCV, UMR 5168, Grenoble F-38054, France; 5 CEA, Direction des Sciences du Vivant, BIG, LPCV, Grenoble F-38054, France; 6 INRA, LPCV, Grenoble F-38054, France

**Keywords:** MIR166, Organizing center, Phyllotaxis, Stem cells, WUSCHEL

## Abstract

The shoot apical meristem (SAM) of angiosperm plants is a small, highly organized structure that gives rise to all above-ground organs. The SAM is divided into three functional domains: the central zone (CZ) at the SAM tip harbors the self-renewing pluripotent stem cells and the organizing center, providing daughter cells that are continuously displaced into the interior rib zone (RZ) or the surrounding peripheral zone (PZ), from which organ primordia are initiated. Despite the constant flow of cells from the CZ into the RZ or PZ, and cell recruitment for primordium formation, a stable balance is maintained between the distinct cell populations in the SAM. Here we combined an in-depth phenotypic analysis with a comparative RNA-Seq approach to characterize meristems from selected combinations of *clavata3* (*clv3*), *jabba-1D* (*jba-1D*) and *erecta* (*er*) mutants of *Arabidopsis thaliana*. We demonstrate that CLV3 restricts meristem expansion along the apical-basal axis, whereas class III HD-ZIP and ER pathways restrict meristem expansion laterally, but in distinct and possibly perpendicular orientations. Our *k*-means analysis reveals that *clv3*, *jba-1D/+* and *er* lead to meristem enlargement by affecting different aspects of meristem function; for example, *clv3* displays an increase in the stem cell population, whereas *jba-1D/+ er* exhibits an increase in mitotic activity and in the meristematic cell population. Our analyses demonstrate that a combined genetic and mRNA-Seq comparative approach provides a precise and sensitive method to identify cell type-specific transcriptomes in a small structure, such as the SAM.

## Abstract (precis)

**Summary:** Three pathways converge to regulate the balance between meristem size, morphology and organization in the *Arabidopsis* shoot apical meristem.

## INTRODUCTION

In plants, all above-ground organs develop post-embryonically from a small group of pluripotent stem cells that reside at the shoot tips in a highly organized structure called the shoot apical meristem (SAM). The SAM can be divided into three functional zones. The central zone (CZ) harbors the self-renewing stem cells that are characterized by low mitotic activity, and their descendants are continuously displaced downward into the interior rib zone (RZ) to further contribute to vascular tissue and stem structures, or toward the surrounding peripheral zone (PZ) (Schoof et al., 2000; Steeves and Sussex, 1989). Cells at the PZ divide at a faster rate and provide the founder cells for leaf or flower primordia that initiate at the flank of the SAM in a precise positioning termed phyllotaxis (Traas, 2013). To ensure proper stable development despite the constant flow of cells from the CZ into the RZ and PZ and their subsequent differentiation and recruitment for primordium initiation, the relative ratio of cells in each functional domain must be maintained. Consequently, the homeostasis between cell proliferation and differentiation is balanced by a complex genetic network that involves hormones, receptor kinase pathways, transcription factors (TFs), small RNAs and chromatin regulators (Sablowski, 2009; Sun and Ito, 2015; Williams and Fletcher, 2005).

At the core of this network is the *WUSCHEL* (*WUS*) gene, which is expressed in the organizing center (OC) at the lower part of the CZ, just below the stem cell reservoir, and encodes a TF that specifies stem cell fate in the overlying cells (Gross-Hardt and Laux, 2003; Laux et al., 1996; Mayer et al., 1998). *WUS* expression is partly restricted by a spatial negative-feedback loop, in which WUS activates the transcription of the *CLAVATA3* (*CLV3*) ligand-encoding gene in the stem cells, and CLV3 in turn restricts *WUS* expression to the OC (Brand et al., 2000; Schoof et al., 2000). Cells that cross the boundary defined by CLV function establish the founder cells for primordia initiation (Schoof et al., 2000). Accordingly, the CZ in *clv3* mutants is dramatically increased in size, leading to enlarged meristems that produce an excess of organ primordia along their periphery (Clark et al., 1995; Fletcher et al., 1999; Szczesny et al., 2009).

Members of the class III homeodomain-leucine zipper (HD-ZIP III) TFs also affect SAM homeostasis (Green et al., 2005; Prigge et al., 2005). In *jabba-1D* (*jba-1D*) plants, overexpression of *MIR166g* causes a decrease in the transcript levels of three HD-ZIP III genes: *PHABULOSA* (*PHB*), *PHAVOLUTA* (*PHV*) and *CORONA* (*CNA*), which leads to expansion of the *WUS* expression domain, resulting in an enlarged SAM (Williams et al., 2005). We previously showed that reduction in ERECTA (ER) kinase receptor-like function enhances the *jba-1D* meristem phenotype, so that the double mutant exhibits extremely enlarged meristems with altered phyllotaxis (Mandel et al., 2014). We hypothesized that the increased surface of *jba-1D/+ er-20* meristems provides adequate distance to allow several auxin maxima, and therefore several organ primordia, to develop simultaneously (Mandel et al., 2014).

Here we combined an in-depth phenotypic analysis with a comparative RNA-Seq approach to characterize and compare meristems from selected single, double and triple mutant combinations of *clv3*, *jba-1D/+* and *er*, which display a gradient of vegetative SAM enlargement. We demonstrate that meristem size determines phyllotaxis pattern. We show that *clv3*, *jba-1D/+* and *er* lead to meristem enlargement by affecting different aspects of meristem function and that the three pathways restrict meristem expansion in distinct directions. Moreover, our bioinformatics analyses demonstrate that the strategy of using different genetic backgrounds and an mRNA-Seq comparative approach facilitates the very sensitive identification of cell-specific gene expression in a small and embedded structure such as the shoot apex.

## RESULTS

### Meristem size regulates phyllotaxis

**Fig. 1.**
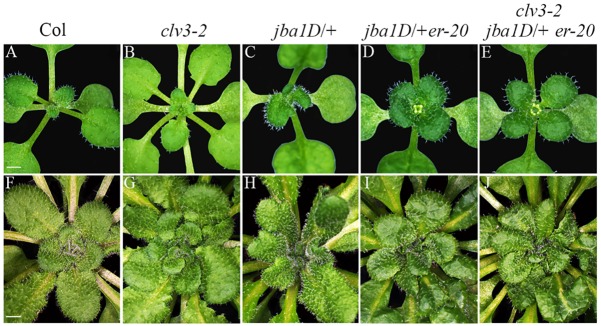
**Genotypes exhibiting increasing meristem size show altered phyllotactic patterns.** Sixteen-day-old *A. thaliana* seedlings grown under long days (A-E) and 45-day-old plants grown under short days (F-J) of Col-0 (A,F), *clv3-2* (B,G), *jba-1D*/+ (C,H), *jba-1D*/+ *er-20* (D,I), *clv3-2 jba-1D*/+ *er-20* (E,J). Col-0 exhibits a typical spiral phyllotactic pattern, whereas *clv3-2 jba-1D*/+ *er-20* exhibits a whorled phyllotactic pattern. Scale bars: 1 mm in A-E; 2 mm in F-J.

We previously reported that the CLV, HD-ZIP III and ER pathways regulate the WUS-dependent inflorescence and floral meristem activities in parallel (Landau et al., 2015; Mandel et al., 2014). Interactions between CLV and ER in regulating shoot and floral meristem, and between CLV and HD-ZIP III in regulating WUS-independent meristem activity, were also reported by others (Durbak and Tax, 2011; Lee and Clark, 2015). To test how these pathways interact to control the vegetative SAM activities, we analyzed seedlings of wild-type Col-0, *clv3-2* and *jba-1D/+* single mutants, a *jba-1D/+ er-20* double mutant, and a *clv3-2 jba-1D/+ er-20* triple mutant. Seedlings of these genotypes grown for 16 days under long-day conditions exhibited a gradual alteration in their phyllotactic pattern. In all genotypes, the first pair of true leaves emerges opposite to each other. However, the expected transition to a spiral phyllotaxis pattern seen in Col-0 (Fig. 1A) is altered in the *clv3-2* mutant to a decussate pattern, in which the two primordia that initiate opposite each other at the same level are perpendicular to the successive pair of primordia (Bartlett and Thompson, 2014) (Fig. 1B). The *jba-1D/+* mutant shows less obvious decussate phyllotaxis (Fig. 1C), although this is difficult to determine due to the curled-leaf phenotype. However, 59 out of 100 of the *jba-1D/+ er-20* seedlings and 64 out of 100 of the *clv3-2 jba-1D/+ er-20* triple mutant exhibit whorled phyllotaxis, in which four leaves are of similar size, suggesting that they developed simultaneously at the same level (Fig. 1D,E). Growing the plants for 45 days under short-day conditions, we observed enhancement of the altered phyllotaxis phenotypes (Fig. 1G-J). Whereas Col-0 plants still exhibited a perfect spiral pattern, the number of leaves emerging simultaneously increased in all mutant genotypes, most dramatically in the triple mutant. The CLV3, HD-ZIP III and ER pathways have all been shown to regulate meristem size (Chen et al., 2013; Clark et al., 1995; Fletcher et al., 1999; Prigge et al., 2005; Uchida et al., 2013), and hence the distinct alteration in phyllotaxis between genotypes and between time points might result from a gradual increase in meristem size that can provide sufficient space to allow the concurrent initiation of multiple leaf primordia.

**Fig. 2.**
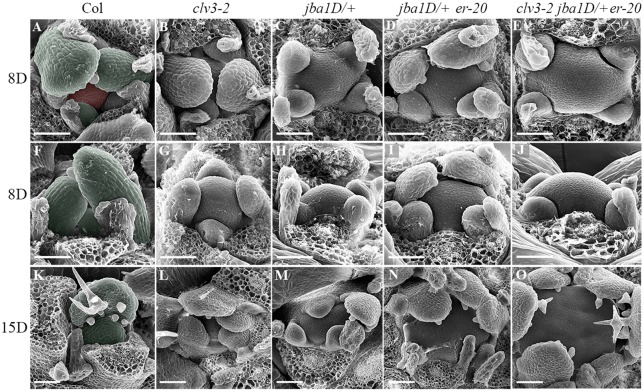
**Mutants with increased meristem size exhibit altered phyllotactic patterns.** SEM images of 8-day-old seedlings showing top (A-E) and side (F-J) view and of 15-day-old seedlings (K-O) of five genotypes with increased meristem size. Typical meristems are shown from among five analyzed per genotype. The meristem sizes of the *jba-1D*/+ single mutant, *jba-1D*/+ *er-20* double mutant and *clv3-2 jba-1D*/+ *er-20* triple mutant gradually increase as the plant grows, with 15-day-old seedlings having much larger meristems than wild type. (A,F,K) Meristem and leaf primordia are false-colored red and green, respectively. Scale bars: 50 μm.

To examine further the effect of meristem size on phyllotaxis, we performed scanning electron microscopy (SEM) analyses of 8- and 15-day-old seedlings of the different genotypes (Fig. 2). The images shown are of typical meristems chosen from among five meristems analyzed per genotype and time point. The SEM images clearly demonstrate the gradient in meristem size from single through double to triple mutants, indicating that the CLV3, HD-ZIP III and ER pathways act in parallel to regulate vegetative meristem size. In addition, the SEM images confirm the initiation of primordia at the same level and therefore confirm the designated phyllotaxis. The increase in meristem size correlates with the alterations in phyllotaxis, which are enhanced after 15 days in all genotypes except for *jba-1D/+*. For example, in *clv3-2*, two leaf primordia emerge in a decussate phyllotaxis in 8-day-old seedlings (Fig. 2B,G), but at 15 days the meristem produces three and sometimes four primordia simultaneously (Fig. 2L).

### Distinct effects of CLV3, HD-ZIP III and ER on meristem shape and size

**Fig. 3.**
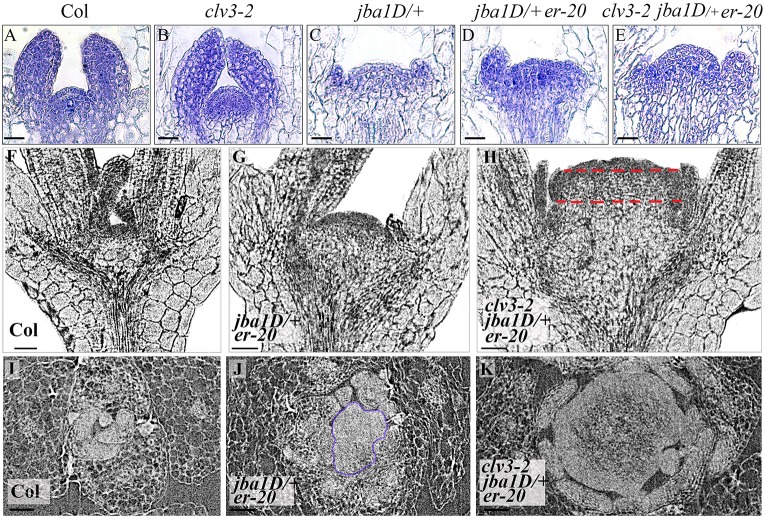
**Mutants with disturbed meristem homeostasis display a gradual increase in meristem size.** (A-E) Longitudinal sections of 8-day-old seedlings and (F-K) X-ray micro-CT sections of 15-day-old seedlings showing (F-H) longitudinal and (I-K) transverse sections. There is a correlation between increased meristem size and the number of leaf primordia. The dashed red lines highlight the shift to a rectangular meristem morphology in the triple mutant. Reconstructed serial sections for 15-day-old seedlings are presented as Movies 1-8. Scale bars: 50 μm.

Longitudinal sections of 8-day-old seedlings further demonstrate the gradual increase in meristem size between the different genotypes, which correlates with the changes in phyllotaxis, such that the triple mutant exhibits the largest meristem (Fig. 3A-E). A striking finding was the distinct nature of the meristem enlargement. Whereas the *clv3-2* meristem expands preferentially in the apical-basal direction (i.e. it is more convex) (Fig. 3B), the *jba-1D/+* and *jba-1D/+ er-20* meristems expanded along the central-to-peripheral axis (radially or laterally) (Fig. 3C,D), and the triple-mutant meristem expanded in both the apical-basal direction and laterally. This suggests that the CLV3 pathway affects meristem shape and organization in a fundamentally different manner than the HD-ZIP III and ER pathways.

To assess the possibility of a temporal increase in meristem size, we used X-ray micro-computed tomography (micro-CT) of 15-day-old seedling meristem from Col-0, *jba-1D/+ er-20* and *clv3-2 jba-1D/+ er-20* mutants (Fig. 3F-K). This technique enabled us to examine both longitudinal (Fig. 3F-H) and transverse (Fig. 3I-K) sections from individual intact meristems, and to generate three-dimensional (3D) reconstructed images (Fig. S1) and movies. In Col-0, the meristem sizes of 8- and 15-day-old seedlings appear to be similar (Fig. 3A,F), indicating that meristem homeostasis is maintained during the additional 7 days of growth. This concurs with the temporally unaffected spiral phyllotaxis in Col-0 (Fig. 1F, Fig. 3I). However, a minor increase in size of the *jba-1D/+ er-20* double-mutant meristem and a dramatic enlargement of the triple-mutant meristem (Fig. 3B,D,G,H) indicate that once the homeostasis is impaired, the imbalance enhances over time. The 15-day-old *clv3-2 jba-1D/+ er-20* seedling exhibits a much wider and taller meristem than *jba-1D/+ er*-*20* and Col-0. Moreover, a 3D view of Col-0 and the triple mutant (Movies 1 and 2) strongly emphasizes the dramatic increase in the triple-mutant meristem in all three dimensions (*x*, *y* and *z* axes), and shows that the meristem acquires a cylindrical shape. Movies of reconstructed serial longitudinal sections (RCSs) (Movies 3-5) reveal that the typical dome-shaped meristem morphology observed in Col-0 and the *jba-1D/+ er-20* mutant shifts toward a rectangular-like shape in the triple mutant, in which the basal and top meristem widths are similar (Fig. 3H, red dashed lines). However, transverse sections and RCS movies (Fig. 3I-K, Movies 6-8), which provide a top view, reveal that the 15-day-old *clv3-2 jba-1D/+ er-20* meristem is circular, indicating that at 15 days the triple-mutant SAM expands radially, equally in all central-to-peripheral axes, forming an isodiametric rather than elongated meristem (fasciated). These sections also highlight the massive increase in meristem size that occurs when all three pathways are reduced.

**Fig. 4.**
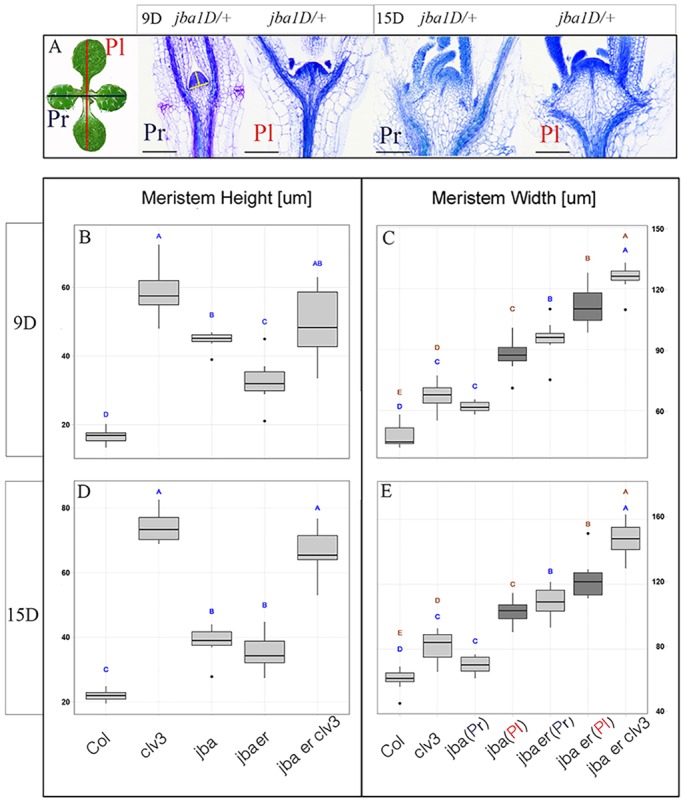
**Vegetative SAM width and height in wild-type and mutant seedlings.** Measurements of meristem width and height were made on median longitudinal section through 9- and 15-day-old seedlings. (A) For two genotypes, namely *jba-1D/+* and *jba-1D/+ er-20*, longitudinal sections were prepared in two orientations: parallel to the cotyledons (red line, Pl) or perpendicular to the cotyledons (blue line, Pr). An example for both orientations in 8- and 15-day-old seedlings is shown for *jba-1D/+* (meristem size appears to be indistinguishable between the two orientations in other genotypes). Meristem width and height were measured in ImageJ as shown by the yellow lines. Scale bars: 100 µm. (B-E) Box plot representations of data derived by image analysis of meristem height (B,D) and width (C,E) of 9- and 15-day-old seedlings. Black center lines show the median; box limits indicate the 25th and 75th percentiles; whiskers extend to 5th and 95th percentiles, outliers are marked by dots; for all measurements *n*=10 (the data are given in Table S1). Representative meristem sections for the two time points for all genotypes are shown in Figs S2 and S3. Significant differences (*P*<0.05, non-parametric multi-comparison Steel-Dwass test) are indicated by different lowercase letters above the box. In C and E the analysis was performed separately for parallel (blue letters) and for perpendicular (brown letters) sections.

To quantify the changes in meristem width and height, we measured sections of ten meristems from each genotype at 9 and 15 days (Fig. 4, Figs S2 and S3). Genotypes with a *clv3-2* background exhibit significantly taller meristems than other genotypes in both 9- and 15-day-old seedlings (Fig. 4B,D). A gradual increase in meristem width was observed, in which the triple mutant exhibits the widest meristem (Fig. 4E,C). The *jba-1D/+* and *jba-1D/+ er-20* meristems expand laterally (Fig. 3C,D), yet in the SEM images (*n*=5) the *jba-1D/+* meristem is elongated whereas the *jba-1D/+ er-20* meristems appear to be more circular (Fig. 2C,D). Longitudinal sections provide two-dimensional (2D) information. Therefore, to be more precise we sectioned these genotypes in two orientations: parallel (Pl) or perpendicular (Pr) to the cotyledons as described in Fig. 4A. As expected, the widths of *jba-1D/+* and *jba-1D/+ er-20* meristems differ depending on the orientation of sectioning. However, whereas *jba-1D/+* shows a difference between average meristem widths with 70 µm for Pr and 103 µm for Pl at 15 days, the difference in *jba-1D/+ er-20* is minor with 109 µm for Pr and 122 µm for Pl (Fig. 4E, Fig. S3, Table S1). This provides evidence for the elongated meristem in *jba-1D/+*, in which the meristem is narrow in one direction and wide in the other, and suggests that the *jba-1D/+ er-20* meristem is more circular. Adding *er-20* to *jba-1D/+* leads to meristem enlargement, but most striking is the result obtained when analyzing the widths of the meristems separately for each orientation: the increase in width in the Pr orientation is much greater (70 µm for *jba-1D/+* to 109 µm for *jba-1D/+ er-20*) than in the Pl orientation (103 µm to 122 µm) (Fig. 4E, Table S1). Altogether, this suggests that in *jba-1D/+* the meristem expands laterally in one direction and that the addition of *er-20* leads to expansion in the perpendicular direction. It also indicates that the direction of meristem expansion is associated with the position of the cotyledons, a phenomenon that will provide an exciting challenge for future investigation.

**Fig. 5.**
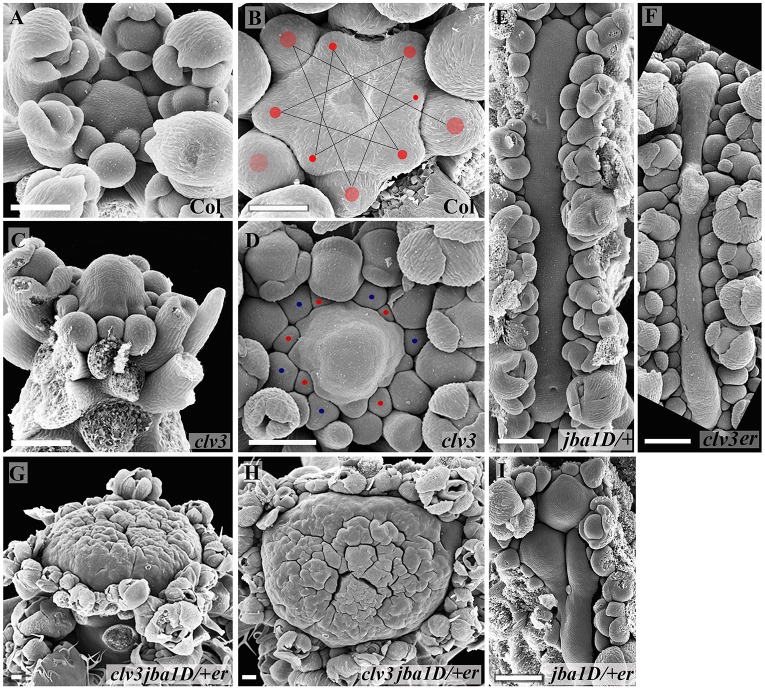
**Reductions in CLV3, HD-ZIP III and ER functions differentially affect meristem morphology.** SEM images of inflorescence meristems (IMs) 24 days after bolting reveal different sizes and shapes in the different genotypes. (A,B) Col-0 IM displaying a spiral phyllotactic pattern. (C,D) *clv3-2* IM (in Col-0 background) displaying a taller structure and altered phyllotaxis. (E) *jba-1D*/+ fasciated IM. (F) *clv3-2 er-20* IM exhibiting a long and narrow meristem. (G,H) *clv3-2 jba-1D*/+ *er-20* IM displaying enlargement in all directions. (I) Adding *er-20* to *jba-1D*/+ causes the meristem to become wider and to split. (A,C,G) Side view; (B,D-F,H,I) top view. The images are of typical meristems from among five meristems analyzed per genotype. Scale bars: 100 µm in A,C-I; 50 µm in B.

SEM analysis of inflorescence meristems (IMs) at 24 days after bolting further highlights the fundamental differences in the regulation of meristem morphology by each of the pathways (Fig. 5). Images are shown for typical meristems chosen from among five meristems analyzed per genotype. In the IM, the perfect spiral phyllotaxis of Col-0 (Fig. 5A,B) is altered in *clv3-2*, in which five to six primordia initiate at the same level (Fig. 5D). The *clv3-2* IMs also exhibit a dramatic increase in height (Fig. 5C, Fig. S4B,C); however, width and length measurements of top view SEM images of IMs (*n*=8) sampled 14 days after bolting (in the case of circular meristem it is the diameter), reveal a minor increase in *clv3-2* diameter compared with Col-0 meristems (Fig. 5A, Fig. S5). This phenotype demonstrates that in *clv3-2* the IM also expands preferentially in the apical-basal direction. Although the *clv3-2* IM is much taller, flower primordia are formed only at the flank of the meristem base, with no organ initiating on the upper part of the dome. In line with previous reports proposing that loss-of-function mutations in *CLV3* result in a dramatic increase in the stem cell population (Fletcher et al., 1999; Laufs et al., 1998; Schoof et al., 2000), we suggest that most of the upper dome section is the result of an increase in the stem cell population, which retains a pluripotent fate, and in CZ size, thus preventing cell differentiation and primordium initiation.

By contrast, the *jba-1D/+* vegetative meristem expands laterally (Fig. 2C,M) and the IM is extremely fasciated (Fig. 5E, Fig. S4). The *jba-1D/+* IM exhibits the largest ratio between length and width of the IM (17.38, Fig. S5C). This indicates that the HD-ZIP III genes do not affect meristem growth equally in all directions but rather restrict expansion to a specific lateral orientation. Adding *er* to *jba-1D/+* further increases SAM size but the meristem expands along the axis perpendicular to that of the elongated *jba-1D/+* meristem, shifting the vegetative meristem toward a more symmetrical dome (Fig. 2D,I,N) and the IM to a less elongated but wider form than that of *jba-1D/+*. Although, as was previously reported in some *jba-1D/+ er-20* plants (Mandel et al., 2014), and in the triple mutant the IMs split into small units (Fig. 5), the lateral organs form only at the periphery of the entire IM structure, and therefore we refer to it as one meristem. The wider IM phenotype supports the hypothesis of the existence of *x* and *y* axes in the presumably symmetrical dome-shaped meristem, and suggests that the HD-ZIP III and ER pathways restrict meristem enlargement laterally in distinct and possibly perpendicular orientations.

Within genotypes the IMs appear to be very similar and show little variability, except for *jba-1D/+ er-20*, where IMs vary in shape and size (Figs S4 and S5). Thus, we aimed to explore *ER* regulation outside of the *jba-1D/+* background. *ER* loss-of-function does not lead to obvious defects in meristem regulation, possibly owing to redundancy (Uchida et al., 2013). To further investigate the involvement of *ER* in the regulation of lateral expansion of the meristem, we analyzed the effect of *er* on the *clv3-2* IM (Fig. 5F, Fig. S4F). Amazingly, *er* transforms the tall, radially symmetrical dome-shaped meristem of *clv3-2* into an extremely narrow, elongated meristem with numerous flower primordia emerging simultaneously, indicating that *ER* restricts meristem lateral expansion.

Further support for the differential directions of expansion is provided by the shape of the triple-mutant meristem. The *jba-1D/+* and *clv3-2 er-20* IMs are both fasciated (Fig. 5E,F). Since the three pathways act in parallel, one would expect that in a cross between the two mutants *jba-1D/+* and *clv3-2 er-20* they would act additively or synergistically and the result will be an extremely elongated/fasciated IM meristem. However, the *clv3-2 jba-1D/+ er-20* triple-mutant IM exhibits an enormously enlarged meristem of remarkably isodiametric shape (Fig. 5H, Fig. S4). The ratio of 1.07 between maximal length and maximal width further demonstrates the circular shape of the IM. Simultaneous growth in two directions perpendicular to each other might lead to a radial shape. Therefore, the radial symmetry of the triple-mutant meristem reinforces the idea of the HD-ZIP III and ER pathways restricting meristem outgrowth in perpendicular lateral directions. Testing this hypothetical scenario constitutes a future challenge.

### Genome-wide analysis of gene expression in the vegetative meristem

SAM enlargement can be due to enlargement of the CZ, the PZ, or both, as a result of an increase in the number of cells that acquire stem cell identity or an increase in the mitotic activity at the PZ, a decrease in cell transition from CZ to PZ, or a decrease in the incorporation of cells into primordia (Pautler et al., 2015; Schoof et al., 2000). Our phenotypic analyses led us to hypothesize that *clv3*, *jba-1D/+* and *er* lead to meristem enlargement by affecting different aspects of meristem function. To test this, we performed mRNA-Seq analysis on pools of meristem-enriched tissues from 45 seedlings that included leaf primordia, similar to those shown in Fig. 2, collected from each of the genotypes as described in Fig. S6 at 8 and 15 days of growth.

**Fig. 6.**
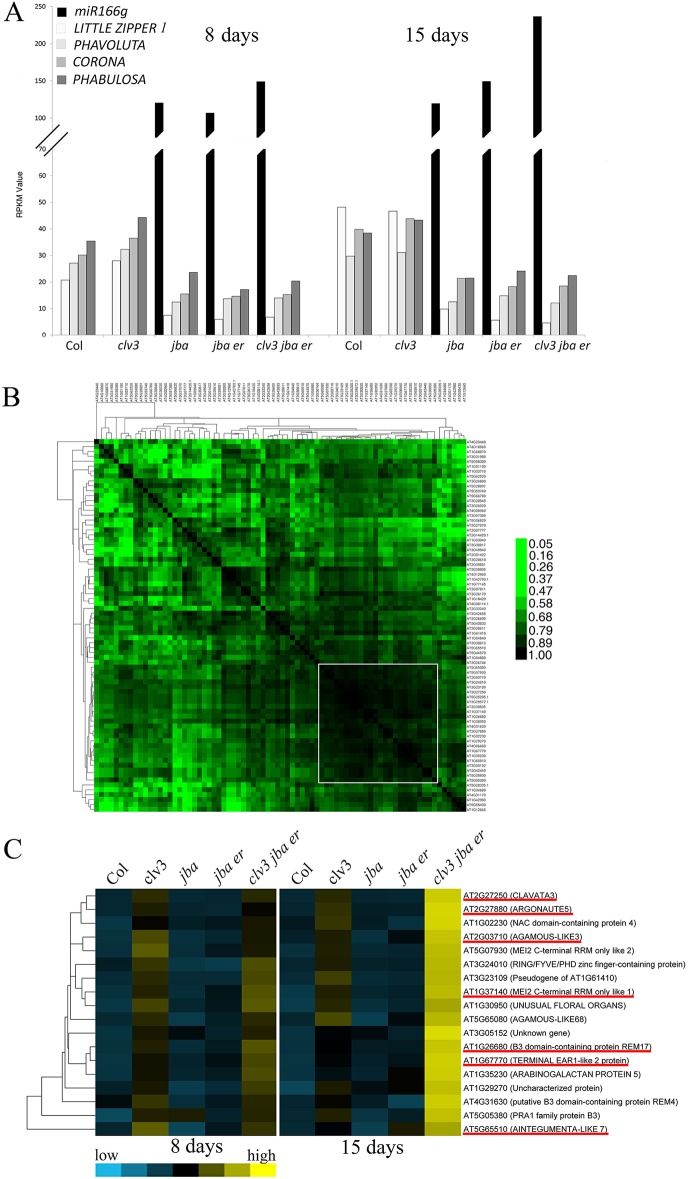
**Expression** **profiles of selected genes in the meristem of wild-type and mutants validate the mRNA-Seq analysis.** (A) RPKM values of *MIR166g*, its HD-ZIP III targets and *ZPR1*, showing a high level of *MIR166g* and a low level of its targets in genotypes with the *jba-1D/+* background, validating the mRNA-Seq analysis. (B) Correlation matrix for the expression of genes in cluster 687. The correlation coefficients for pairs of genes were subjected to two-way hierarchical clustering using Cluster Gene. The white square marks the subset that includes *CLV3*, which is discussed in the text. (C) Heat map of selected genes co-expressed with *CLV3* from cluster 687 (GEO accession GSE79839). Genes shown to be expressed exclusively in the CZ are underlined in red (Yadav et al., 2009). The heat map was produced by clustering the normalized values using the hierarchical clustering algorithm implemented in Gene Cluster (for details see the supplementary Materials and Methods).

The experiment was performed with one replicate owing to the laborious work involved in the collection of meristems. Validation of the results was achieved by examining the expression levels of previously reported genes and by reverse transcription quantitative real-time PCR (RT-qPCR). On average, 40 M reads were mapped to the genome (Table S2) and the expression levels of all annotated genes (TAIR10, https://www.arabidopsis.org/) in each sample, expressed as reads per kilobase of transcript per million sequenced reads (RPKM), was calculated (see GEO accession number GSE79839). To validate our transcriptomic analysis, we first examined the expression of the *MIR166g* (*AT5G63715*) precursor previously shown to be upregulated in *jba-1D*/+ and the expression of three of its HD-ZIP III targets, namely *PHB*, *PHV* and *CNA*, previously shown to be downregulated in *jba-1D/+* (Williams et al., 2005). At both time points, *MIR166g* transcripts were undetectable in Col-0 and *clv3-2* meristems (RPKM of 0), but reached RPKM values between 106 and 236 in the meristems of all mutant combinations in the *jba-1D/+* background (Fig. 6A)*.* This pattern was specific to the *MIR166g* precursor and was not observed in any of the other eight *MIR165* or *MIR166* genes. The three HD-ZIP III members, as well as *LITTLE ZIPPER 1* (*ZPR1*), which requires HD-ZIP III function for expression (Wenkel et al., 2007), were all downregulated in mutants with a *jba-1D/+* background, as compared with Col-0 and *clv3-2* in both 8- and 15-day-old meristems. The mRNA-Seq data were also validated by RT-qPCR performed on these genes and others (see below), using RNA obtained in an independent experiment, all of which showed similar results to those of the mRNA-Seq (Fig. S7).

Differential expression of *CLV3* further validated our analysis. The *CLV3* gene is a well-established marker for stem cells at the tip of the SAM (Aggarwal et al., 2010; Schoof et al., 2000), and *CLV3* is expressed in *clv3-2* meristems as the *clv3-2* allele is known to carry a breakpoint in the third exon that does not prevent its transcription (Fletcher et al., 1999; Hobe et al., 2003). We and others (Fletcher et al., 1999; Laufs et al., 1998; Schoof et al., 2000) have proposed that meristem enlargement in *clv3* mutants is the result of an increase in the stem cell population. Therefore, shifting the ratio between cell populations in the SAM towards more stem cells, we expected to see a high level of *CLV3* transcripts in mutants with the *clv3-2* background. Indeed, the RPKM values were 49 and 47 in *clv3-2*, and 47 and 124 in *clv3-2 jba-1D/+ er-20*, for 8- and 15-day-old meristems, respectively, whereas all other samples showed values in the range of 1.3 to 3.2 (GEO accession GSE79839). Furthermore, to correlate gene expression patterns with phenotypes and meristem functions, a *k-*means (*k*=1000) clustering algorithm was used to assign genes to expression clusters. Out of the 76 genes that were highly correlated in expression with that of *CLV3* and assigned to the same cluster (cluster 687; GEO accession GSE79839), seven were previously shown by *in situ* hybridization to be expressed exclusively in the CZ (Yadav et al., 2009). These seven genes validated our clustering analysis and suggest that our mRNA-Seq analysis identified many candidate stem cell-specific genes for future validation. To identify a more reliable core set of genes related to stem cell function, we further calculated the correlation coefficients for all possible gene pairs in cluster 687 and clustered the genes based on the Euclidean distance of their correlation coefficients using a hierarchical clustering algorithm. The result, represented as a heat map (Fig. 6B), allowed us to select a group of 23 genes that were most highly correlated with the *CLV3* pattern of expression. The relative expression of 18 out of these 23 genes (five were omitted owing to their low RPKM values) in the five genotypes at two time points is illustrated as a heat map (Fig. 6C). The high relative expression levels of these genes in *clv3* and *clv3-2 jba-1D/+ er-20* provides further evidence for a high proportion of stem cells in *clv3* meristems, indicating that *clv3* leads to meristem enlargement by increasing the number of cells specified as stem cells. Furthermore, whereas in *clv3* these 18 genes showed similar expression values in 8- and 15-day-old meristems, in the triple mutant the expression value was much higher in 15-day-old versus 8-day-old meristems (intense yellow in the heat map in Fig. 6C), consistent with the phenotypic enhancement (Fig. 1E,J, Fig. 2E,O, Fig. 3E,H). This strongly supports the idea of a gradual increase in the imbalance of homeostasis when the three pathways regulating meristem function are impaired.

### ER regulates mitotic activity in the PZ

The relatively low expression level of *CLV3* and its co-expressed genes in *jba-1D/+* and *jba-1D/+ er-20* (Fig. 6C, Fig. S7; GEO accession GSE79839) suggests that in both mutants the increase in meristem size is either proportional for all zones, such that the *CLV3* expression level is similar to that seen in Col-0, or results from an increase in the meristematic cell population outside of the CZ that does not express *CLV3*. Another possibility is an increase in the number of leaf primordia, leading to dilution of *CLV3* mRNA.

**Fig. 7.**
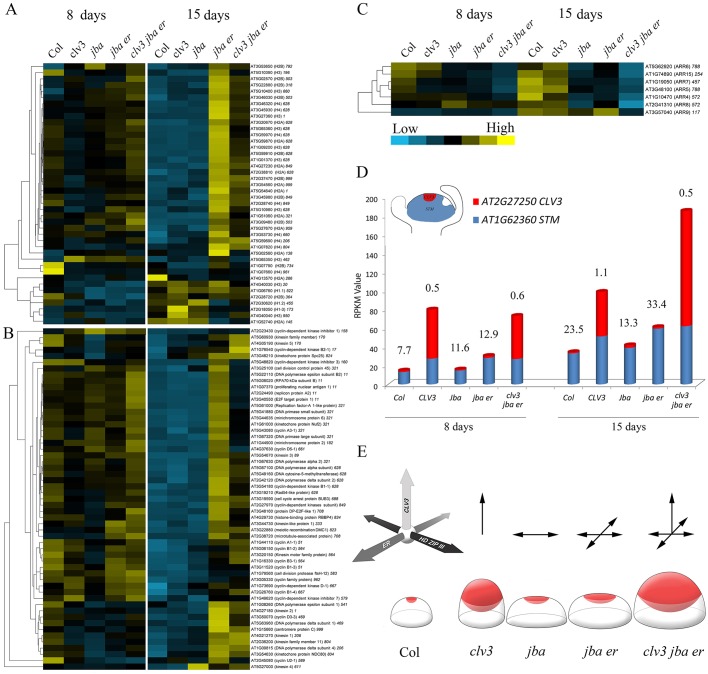
**The differential characteristics of meristems from** **CLV3, HD-ZIP III and ER pathway mutants.** (A-C) Heat maps of expression levels of (A) histones (Table S3), (B) cell cycle genes (Table S4) and (C) *ARR* genes in meristems of the five genotypes from 8-day-old and 15-day-old seedlings (all color-coding as in C). (D) RPKM values of *STM* (blue) and *CLV3* (red) genes. The RPKM value of *STM* divided by that of *CLV3* is indicated above each bar. (E) Model for differential regulation of meristem structure by the CLV3, HD-ZIP III and ER pathways. We propose that *CLV3* restricts meristem expansion along the apical-basal axis, whereas the HD-ZIP III and ER pathways restrict meristem expansion laterally, but in distinct and possibly perpendicular orientations. Mutants with a reduction in one, two or all three pathways exhibit meristem expansion accordingly.

The cell division rate in the CZ is lower than at the meristem periphery (Cockcroft et al., 2000; Geier et al., 2008; Grandjean et al., 2004; Laufs et al., 1998). To gain insight into the proliferation rate in the meristem of the different genotypes, we analyzed the expression patterns of mitotic activity marker genes. Histones are the primary protein components of chromatin and their transcription is tightly coupled to DNA replication during S phase of the cell cycle (Takayama and Toda, 2010); as such, they serve as a marker for cell division activity (Geier et al., 2008; Meshi et al., 2000). The *k*-means analysis, presented as a heat map (Fig. 7A), reveals that out of the 42 histone genes that showed expression in this mRNA-Seq analysis, 11 genes corresponding to the four core histones were assigned to cluster 628 (Table S3; GEO accession GSE79839), which is also enriched with many genes involved in cell division. To categorize the functions of the 52 genes assigned to cluster 628, we searched for significantly enriched gene ontology (GO) terms using singular enrichment analysis, as implemented in agriGO (see the Materials and Methods). Some GO terms representative of those enriched in cluster 628 were ‘DNA-dependent DNA replication' (GO:0006261; *P*=1.37×10^–7^), ‘cell cycle process' (GO:0022402; *P*=6.87×10^–8^), ‘chromosome organization' (GO:0051276; *P*=8.28×10^–14^) and ‘cell proliferation' (GO:0008283; *P*=2.08×10^–7^). This suggests that the *k-*means analysis precisely identified a set of genes that are co-expressed specifically in a group of cells exhibiting high mitotic activity.

Three distinct patterns were evident in the heat map (Fig. 7A). First, the differential expression patterns of all histones between the two time points. Second, a group of seven histones at the lowest part of the heat map exhibits an expression pattern opposite to that of the other histones. Accordingly, their relative expression level is low in the 8-day-old meristems and high in the 15-day-old meristems. Interestingly, the three genes encoding histone H1, which links nucleosomes into higher order structures, appear in this group. Third, the remarkable differential expression between genotypes in the 15-day-old meristems. The highest relative expression level is observed in *jba-1D/+ er-20* meristems, indicating high mitotic activity, and implying that *jba-1D/+ er-20* meristems have an enlarged PZ with high cell proliferation rates. Alternatively, a change in the ratio between meristem and leaf primordium numbers, where a high rate of cell division takes place, could also explain this high mitotic activity. However, the SEM images of 8-day-old seedlings reveal, for example, that *jba-1D/+ er-20* has four primordia (Fig. 2D) and that *clv3-2* at 15 days has three young primordia surrounding a small meristem (Fig. 2L), suggesting a high ratio of primordia cells to meristem cells. Yet, both *jba-1D/+ er-20* at 8 days and *clv3-2* at 15 days exhibit relatively low expression of histones and cell cycle genes. The high ratio of leaf primordia to meristem in *clv3-2* at 15 days as compared with *jba-1D/+ er-20* is also clearly demonstrated in all the sections (Fig. S3B,E,F). Therefore, we believe that the relatively high expression of histones and cell cycle genes observed in *jba-1D/+ er-20* is due to an increase in the number of meristematic cells and not to an increase in the ratio of primordia to meristem. The expression pattern of cell cycle genes provides additional support for the low mitotic activity in Col-0, *clv3* and *jba-1D/+*, and for the moderate and high mitotic activity in *clv3-2 jba-1D/+ er-20* and *jba-1D/+ er-20* meristems, respectively (Fig. 7B, Table S4). These analyses demonstrate that adding *er* to *jba-1D/+* leads to an increase in mitotic activity at the meristem. Furthermore, our analysis reveals that Col-0 and *jba-1D/+* exhibit similar expression patterns of stem cell genes (Fig. 6C) and mitotic marker genes (Fig. 7B) in both 8- and 15-day-old meristems, suggesting that the increase in meristem size in *jba-1D/+* is proportional for all zones.

Another interesting pattern of meristem-related gene expression was that of the TYPE-A RESPONSE REGULATOR genes *ARR5*, *ARR6*, *ARR7* and *ARR15* (Fig. 7C), which are negative regulators of cytokinin signaling and meristem size, and are directly repressed by WUS (Leibfried et al., 2005). The triple-mutant meristems showed the lowest level of expression at both time points (GEO accession GSE79839) and the general expression pattern of the *ARR* genes resembled that of the three HD-ZIP III and *ZPR1* genes (Fig. 6A), with lowest expression in mutants of the *jba-1D/+* background. In the *k*-means analysis, *ARR5* and *ARR6* were assigned to cluster 788 together with *ZPR1* and *ZPR4* (GEO accession GSE79839), suggesting a link between HD-ZIP III, *ZPR* and *ARR* genes.

The SHOOT MERISTEMLESS (STM) TF functions in the meristem to prevent the premature incorporation of cells into differentiation pathways. Accordingly, it is expressed in both the CZ and PZ of the meristem and repressed in organ primordia (Gallois et al., 2002; Long et al., 1996). To further investigate the ratio between domains in the meristem, we looked at the ratio of expression levels of the meristematic cell marker *STM* (Geier et al., 2008) and the stem cell marker *CLV3* (Fig. 7D). When dividing the *STM* RPKM value by that of *CLV3*, genotypes with the *clv3-2* background show the lowest ratio at both time points, implying that these meristems harbor a higher proportion of stem cells than wild-type meristem, whereas *jba-1D/+ er-20* exhibits the highest ratio, implying that its meristem harbors a high proportion of meristematic cells. Similar results were obtained in RT-qPCR validation experiments (Fig. S7), consistent with a previous report that *jba-1D/+ er-20* exhibits higher *STM* expression levels than *jba-1D/+* (Mandel et al., 2014). This demonstrates the effect of *er* on *jba-1D/+*, i.e. that of promoting an increase in the meristematic cell population, which exhibits increased mitotic activity. It also suggests that the relatively high expression of histones and cell cycle genes in *jba-1D/+ er-20* is the result of an increase in the meristematic cell population and not of leaf primordia. Altogether, the ratios between *STM* and *CLV3* expression values support our conclusions from the phenotypic and mRNA-Seq analyses regarding meristem organization.

## DISCUSSION

During plant development, the stem cells in the SAM proliferate indeterminately to continuously produce organs, yet the SAM is restricted to a very small and stable dome-shaped structure. In this study, we show that CLV3, HD-ZIP III and ER restrict meristem expansion in distinct directions and affect different aspects of meristem function, thereby regulating meristem size, organization and morphology. Removing all three pathways enhances the gradual temporal shift in meristem homeostasis seen with the removal of only one or two pathways. We also explicitly demonstrate that meristem size and organization determine the phyllotaxis pattern.

It is well established that auxin maxima determine primordium initiation sites at the SAM periphery, and that new auxin maxima can be formed only at a certain minimal distance from existing primordia (Lohmann et al., 2010; Reinhardt et al., 2003; Sassi and Vernoux, 2013). Moreover, it was shown that the L1 layer of the SAM serves as a conduit for auxin transport and that the phyllotactic patterns form on the surface of the SAM (Kierzkowski et al., 2013; Smith et al., 2006). Our results support this idea. For example, *clv3* 8-day-old SAM exhibits a similar width to Col-0 SAM but is taller and more convex, resulting in an increased SAM surface. This allows two leaf primordia to develop simultaneously opposite to each other at the two farthest sites, resulting in a decussate phyllotaxis. Our mRNA-Seq analysis reveals that the ratio between zones in *clv3* meristems is altered toward increased CZ due to an increase in the stem cell population. This is consistent with a computer simulation demonstrating that a shift to a decussate pattern requires a decrease in the peripheral width and an increase in the CZ (Smith et al., 2006).

Loss of all *ER* family genes leads to a flattened meristem with lateral expansion of the *WUS* expression domain (Chen et al., 2013; Uchida et al., 2013). Longitudinal sections of GUS analysis in *jba-1D/+* seedlings also show lateral expansion of the *WUS* domain (Williams et al., 2005), indicating that the ER and HD-ZIP III pathways restrict the expression of *WUS* in cells flanking the OC. However, by analyzing the expression pattern in 2D sections, we cannot determine whether the *WUS* domain expands laterally or radially. Nevertheless, our two-orientation sectioning analysis of *jba-1D/+* indicates that in one orientation the meristem is narrow compared with the perpendicular orientation, suggesting that the *WUS* domain expands laterally. The phenotypic analysis demonstrates that both ER and HD-ZIP III restrict meristem expansion laterally, and therefore a reduction in their function leads to elongated meristems. However, when *jba-1D/+* and *er-20* are combined, both in the *jba-1D/+ er-20* double mutant and the *clv3-2 jba-1D/+ er-20* triple mutant, the meristems are much larger and isodiametric, suggesting expansion in two perpendicular lateral directions. When *clv3* is added to the *jba-1D/+ er-20* mutant, further expansion in the apical-basal axis makes the meristem more cylindrical, indicating expansion in all directions (Fig. 7E).

If members of the HD-ZIP III restrict meristem expansion in a specific lateral direction that differs from the direction of restriction by ER, how are these directions determined and established in the dome-shaped meristem? In *Arabidopsis*, after germination the SAM is a flattened bilaterally symmetric structure, which changes after day 7 to a radially symmetrical dome (Bowman, 1994; Medford et al., 1992). We hypothesize that the lateral directional restrictions by HD-ZIP III and ER are determined during embryo development at the onset of bilateral symmetry formation, simultaneously with cotyledon formation. Once the axes of regulation are established they are stable, regardless of the formation of the radially symmetrical dome-shaped meristem. Although our differential sectioning analysis associates the expansion direction with the cotyledon growth direction, further research is required to test this hypothesis.

The SAM is a small, complex structure composed of many cell types, each expressing a specific set of genes. Revealing the spatiotemporal gene expression patterns at the SAM can provide a better understanding of cell fate specification and of cellular processes at the level of particular cell types. Several methods have been applied to identify cell type-specific gene expression in the SAM, including laser microdissection (Brooks et al., 2009; Ohtsu et al., 2007), fluorescence-activated cell sorting (Yadav et al., 2009) and isolation of nuclei tagged in specific cell types (Deal and Henikoff, 2010). Our mRNA-Seq and *k-*means analyses of meristem-enriched tissue assigned many groups of genes that are known to be co-expressed to the same clusters. Thus, the strategy of using different genetic backgrounds combined with a comparative analysis of mRNA-Seq data provides a sensitive approach for the identification of co-regulated genes expressed at low level in an embedded small structure such as the shoot apex. Our data will serve as a useful resource to dissect spatial regulatory pathways and for the identification of genes acting in the same process or complex, as well as for the construction of gene networks acting in the SAM.

## MATERIALS AND METHODS

### Growth conditions and plant materials

The plant materials used in this study were *Arabidopsis thaliana* Columbia (Col-0), *jabba-1D* (*jba-1D*) (Williams et al., 2005), *clavata3-2* (*clv3-2*) (Clark et al., 1995) and *erecta-20* (*er-20*) (Mandel et al., 2014). Plants were grown under long-day (16 h light/8 h dark) or short-day (8 h light/16 h dark) conditions at 18-22°C in soil or on Murashige and Skoog (MS) plates.

### Microscopy and histology

Whole-plant images were captured using an Olympus SZX7 stereomicroscope. Images of transverse sections were captured using an Olympus IX8 Cell-R inverted microscope. SEM was performed by fixing tissue in methanol as described previously (Talbot and White, 2013) for examination in a JEOL 5410 LV scanning electron microscope. For histological analyses, seedlings were fixed in 4% formaldehyde fixative with 50% ethanol and 5% glacial acetic acid, vacuum infiltration, dehydrated, embedded in paraffin wax, sectioned at 8 μm thickness and stained with Toluidine Blue. SAM images were measured using ImageJ software (NIH). Statistical analysis of all measurements was performed using the non-parametric multi-comparison Steel-Dwass test due to unequal variance between the different genotypes using JMP 12 software (SAS).

### Micro-CT analysis

Seedlings were fixed by formaldehyde-acetic acid vacuum infiltration and dehydrated using an ethanol series. Seedlings in 100% ethanol were then soaked in 2% potassium iodide (IKI) and were scanned using the Micro XCT 400 system (XRadia). Movies of reconstructed serial longitudinal sections (RCS) were made manually.

### Differential expression analysis by mRNA-Seq

SAM-enriched tissues from 8- and 15-day-old seedlings were collected as described in Fig. S6 (each sample comprised 45-50 meristems), and were immediately frozen in liquid nitrogen. Total RNA was isolated using the RNeasy Mini Kit (Qiagen) and used to prepare ten libraries using the TruSeq-RNA Kit (Illumina). Single-end sequencing was performed by multiplexing the libraries in an Illumina HiSeq 2500 system at the Technion Genome Center (Israel). The reads (an average of 40 M per sample at 51 nucleotides long) were quality filtered and trimmed using Trimmomatic 0.32 (see the supplementary Materials and Methods) and then aligned to the TAIR10 version (https://www.arabidopsis.org/) using TopHat v.2.0.12 and Bowtie2 v.2.1.0 (supplementary Materials and Methods). The resulting alignments (Table S2) were quantified with Cuffdiff v.2.2.1 (supplementary Materials and Methods). The gene expression levels (RPKM) were subjected to *k*-means clustering using Gene Cluster 3.0 (de Hoon et al., 2004), with *k=*1000, and ‘correlation (centered)' as the similarity metric. Heat maps of selected genes were visualized using Java Treeview 3.0 as described in the supplementary Materials and Methods.

### Gene ontology analysis

Selected genes were subjected to gene ontology (GO) analysis using the agriGO online tool available at http://bioinfo.cau.edu.cn/agriGO/index.php, selecting the *Arabidopsis thaliana* TAIR10 genome annotation as the background reference, hypergeometric statistical tests with Bonferroni correction, and *P*≤0.05 (Du et al., 2010).
